# Glycine Betaine and β-Aminobutyric Acid Mitigate the Detrimental Effects of Heat Stress on Chinese Cabbage (*Brassica rapa* L. ssp. *pekinensis*) Seedlings with Improved Photosynthetic Performance and Antioxidant System

**DOI:** 10.3390/plants11091213

**Published:** 2022-04-29

**Authors:** Jin Quan, Weiwei Zheng, Meifang Wu, Zhuojun Shen, Jingru Tan, Zewei Li, Biao Zhu, Seung-Beom Hong, Yanting Zhao, Zhujun Zhu, Yunxiang Zang

**Affiliations:** 1 Key Laboratory of Quality and Safety Control for Subtropical Fruit and Vegetable, Ministry of Agriculture and Rural Affairs, Collaborative Innovation Center for Efficient and Green Production of Agriculture in Mountainous Areas of Zhejiang Province, College of Horticulture Science, Zhejiang A&F University, Hangzhou 311300, China; 2020601042036@stu.zafu.edu.cn (J.Q.); zhengww@zafu.edu.cn (W.Z.); wumeifang8@163.com (M.W.); 13967067051@163.com (Z.S.); t1399501017@163.com (J.T.); a460759461@163.com (Z.L.); billzhu@zafu.edu.cn (B.Z.); zhuzj@zafu.edu.cn (Z.Z.); 2 Department of Biotechnology, University of Houston Clear Lake, Houston, TX 77058-1098, USA; dsbhong@yahoo.com; 3 Institute of Vegetables, Zhejiang Academy of Agricultural Sciences, Hangzhou 310021, China; yanting0351@163.com

**Keywords:** glycine betaine, β-aminobutyric acid, Chinese cabbage, heat stress, photosynthesis, antioxidant enzyme activity, osmoprotection

## Abstract

Heat stress is one of the major abiotic factors that limit the growth, development, and productivity of plants. Both glycine betaine (GB) and β-aminobutyric acid (BABA) have received considerable attention due to their roles in stimulating tolerance to diverse abiotic stresses. In order to understand how GB and BABA biostimulants alleviate heat stress in a cool-weather Chinese cabbage (*Brassica rapa* L. ssp. *pekinensis*) plant, we investigated the GB- and BABA-primed heat-stressed plants in terms of their morpho-physiological and biochemical traits. Priming with GB (15 mM) and BABA (0.2 mM) was conducted at the third leaf stage by applying foliar sprays daily for 5 days before 5 days of heat stress (45 °C in 16 h light/35 °C in 8 h dark) on Chinese cabbage seedlings. The results indicate that GB and BABA significantly increased chlorophyll content, and the parameters of both gas exchange and chlorophyll fluorescence, of Chinese cabbage under heat stress. Compared with the unprimed heat-stressed control, the dry weights of GB- and BABA-primed plants were significantly increased by 36.36% and 45.45%, respectively. GB and BABA priming also greatly mitigated membrane damage, as indicated by the reduction in malondialdehyde (MDA) and electrolyte leakage through the elevation of proline content, and increased activity levels of superoxide dismutase (SOD), peroxidase (POD), catalase (CAT), and ascorbate peroxidase (APX). Taken together, GB and BABA have great potential to enhance the thermotolerance of Chinese cabbage through higher photosynthesis performance, osmoprotection, and antioxidant enzyme activity.

## 1. Introduction

Global climate change has led to a worldwide increase in the frequency of high temperature periods in recent years [1]. Scientists predict that global mean annual temperatures will increase by 2.6–4.8 °C by 2081–2100 relative to 1986–2005 [2]. Heat stress is a critical factor that limits plant growth and development, and ultimately diminishes the yield and quality of crops [3,4]. An increase of 1 °C in annual temperature would result in 2.5–16% crop yield reduction [5,6].

Photosynthesis is one of the most sensitive physiological processes in terms of response to high temperature and is often inhibited before other cell functions are impaired by heat [7]. In the photosynthetic apparatus, photosystem II (PSII) is regarded as the most heat-labile component, which primarily limits photochemistry in response to environmental perturbations and stresses, including heat and high light intensity [8,9]. The efficiency of photosynthetic electron transport and ATP synthesis is greatly diminished and coincides with the increased production of reactive oxygen species (ROS), when PSII suffers severe thermal damage [10]. The inhibition of PSII activity usually leads to changes in variable chlorophyll fluorescence (ChlF). Thus, measurements of ChlF related to photosynthesis can shed light on the ability of plants to tolerate environmental stresses, and the extent to which these stresses damage the photosynthetic apparatus [11].

Heat stress disrupts cellular homeostasis due to the generation of an excessive level of reactive oxygen species (ROS), such as superoxide anion (O_2_^−^), hydrogen peroxide (H_2_O_2_), hydroxyl radical (HO^.^), and singlet oxygen (^1^O_2_) [12]. Moreover, plant defense mechanisms are often inadequate to provide sufficient protection. Increasing the ROS detoxification capacity is considered an efficient defense strategy for ameliorating heat stress in plants [13]. This antioxidant defense system comprises ROS-scavenging enzymes, such as catalase (CAT), superoxide dismutase (SOD), peroxidase (POD), and ascorbate peroxidase (APX) [14]. SOD acts as the first line of defense against ROS through the dismutation of O_2_^−^ into H_2_O_2_ and O_2_ in multiple sites, including the chloroplast, cytosol, mitochondria, peroxisome, and apoplast, while POD reduces H_2_O_2_, or organic peroxides, to H_2_O in cell walls and vacuoles [15,16,17]. CAT directly converts H_2_O_2_ into H_2_O and 1/2O_2_, mainly in peroxisomes, whereas APX reduces H_2_O_2_ to H_2_O, using ascorbate as a specific electron donor, in the multiple sites [18].

Although exogenous application of phytohormones, such as abscisic acid, brassinosteroid, cytokinin, salicylic acid, and jasmonic acid, improves plant heat tolerance [19], the use of the phytohormones in the agricultural field is not economically feasible due to their high costs. Use of biostimulants, such as seaweed extract and plant-growth-promoting microorganisms, is assumed to be a more economical and ecofriendly priming approach to improve abiotic stress tolerance, as well as nutrition efficiency, crop yield, and quality traits [20]. It was reported that the application of seaweed extract improved vegetative growth under nutrient-deficient conditions by improving photosynthesis and root growth in tomato plants [21]. Numerous studies suggest that biostimulants can help plants tolerate a variety of stresses, including salinity, heat, and drought, by enhancing the antioxidant system [22,23]. Biostimulants have been classified into six non-microbial and three microbial categories [24]; among the former are protein hydrolysates, which contain mainly peptides and free amino acids [25,26]. Protein hydrolysate-based products can be further divided into two major categories, a mixture of peptides and amino acids of animal or plant origin, and individual amino acids [27].

Glycine betaine (GB), an *N*,*N*,*N*-trimethyl glycine, is naturally found in haloarchaea, bacteria, marine invertebrates, plants, and animals [28,29]. As a zwitterionic quaternary amine and a key osmoprotectant [30,31], GB priming stabilizes the quaternary structure of proteins, including the PSII complex and enzyme activities, as well as maintains the integrity of membranes against the damaging effects of abiotic stresses [25,32,33,34,35]. Moreover, transgenic tomato plants expressing the key genes for GB biosynthesis reportedly displayed significantly enhanced PSII activity, CO_2_ assimilation, heat shock protein level, and thermotolerance [36]. Consequently, it effectively combats heat stress not only by reducing photosynthesis inhibition and excessive ROS accumulation, but also by activating stress-responsive genes under high temperature stress [31,36,37,38,39,40].

β-aminobutyric acid (BABA) is a non-protein amino acid that is naturally present in plants as a defense-priming molecule capable of inducing resistance to a wide range of biotic and abiotic stresses [41]. For instance, BABA priming significantly reduced the damages caused by chilling, heat shock, and salinity by activating antioxidant defense systems [42,43,44]. BABA-induced thermotolerance was accompanied by elevated transcript levels for several transcription factors and DNA binding proteins regulating responses to the stress hormone abscisic acid [45]. In addition, BABA priming conferred faster and stronger resistance against numerous plant pathogens via a variety of defense mechanisms, including physical barriers and biochemical changes [46,47,48]. Both GB and BABA were found to be absorbed by the directly treated leaves, and readily transported throughout the plant [46,49].

Chinese cabbage (*Brassica rapa* L. ssp. *pekinensis*) is a cold season leafy vegetable crop of major economic importance in many countries [50]. Its growth temperature ranges from 7 to 24 °C, but when it is sown early in August at high temperatures (over 30 °C) to harvest in the autumn, especially in East Asian countries [51,52], this usually results in dramatically reduced yield and quality, and even leads to plant death [50,53,54]. Chinese cabbage seedlings, which are more sensitive to unfavorable thermal conditions than more developed plants, can tolerate high temperatures up to 27 °C [55]. Therefore, the development of strategies to enhance the thermotolerance capacity of Chinese cabbage would be valuable to the farming industry. To date, there are very few studies on the use of biostimulants in Chinese cabbage; there is one report on the impacts of biostimulants as a mixture of amino acids and seaweed extract on the growth of *B. rapa* [56]. In this study, we used two amino acid-based GB and BABA biostimulants to evaluate their effects on the morpho-physiological and biochemical traits of *B. rapa* seedlings under heat stress. Our results contribute to the understanding of a physiological and biochemical mechanism for better tolerance to heat stress conferred by GB and BABA. Therefore, it provides a theoretical and practical basis for applying GB and BABA to improve the sustainability of Chinese cabbage cultivation in high-temperature climates.

## 2. Results

### 2.1. Effects of GB and BABA on the Growth of Chinese Cabbage Seedlings under Heat Stress

**Figure 1:**
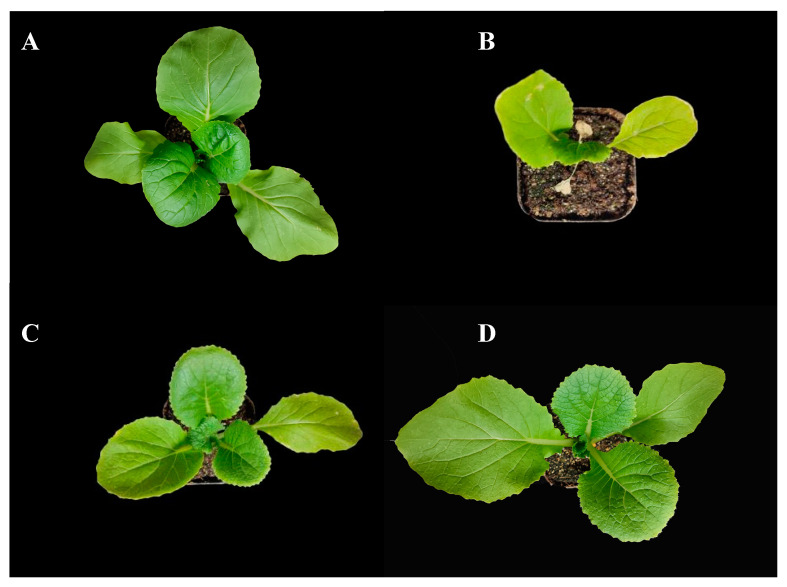
Morphology and growth phenotypes in response to exogenous GB and BABA in *B. rapa* under heat stress. Ctrl (**A**), optimum conditions; HT + Ctrl (**B**), HT + GB (**C**), and HT + BABA (**D**) seedlings were foliar sprayed with water, GB, and BABA, respectively, before exposure to high temperature.

In order to evaluate the effects of GB and BABA, we first assessed the morphological changes and growth parameters. Chinese cabbage seedlings grew vigorously at normal temperature (24 °C). Under heat stress (45 °C), seedlings became stunted and wilted, with some leaves turning yellowish, whereas those plants pretreated with GB or BABA did not wilt. Furthermore, GB- and BABA-primed plants were healthy, with expanded green leaves, when compared to control plants under optimal conditions (Figure 1). No difference in the number of leaves was observed in all of the treatments. The heat-stressed control seedlings showed 39.27% and 44.07% lower fresh weight and dry weight than the control under optimum conditions, respectively. As compared to the heat-stressed control, GB treatment increased fresh weight (25.93%), dry weight (36.36%), leaf length (25.45%), width (8.35%), plant height (17.38%), and hypocotyl diameter (7.11%). BABA application increased fresh weight (34.47%), dry weigh (45.45%), leaf length (36.11%), width (26.98%), plant height (19.60%), and hypocotyl diameter (12.00%) (Table 1). GB and BABA treatment also enhanced the chlorophyll content by 9.75% and 15.45%, respectively (Table 1). However, no significant differences were noted in the vegetative growth parameters, except for leaf width, between GB- and BABA-primed plants under heat stress. GB- and BABA-primed plants displayed markedly decreased biomass and chlorophyll content relative to the control under optimum conditions. These results show that both GB and BABA had positive influence on the morphological parameters and chlorophyll content, though their treatment did not fully restore morpho-physiology to the level of the control plants.

### 2.2. Effects of GB and BABA on MDA, Electrolyte Leakage, and Proline Content in Chinese Cabbage under Heat Stress

**Figure 2:**
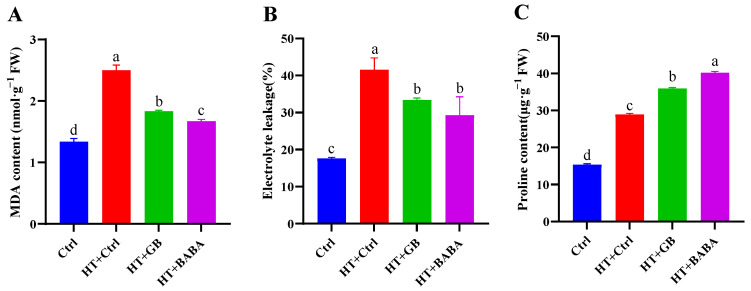
Effects of GB and BABA on MDA content (**A**), electrolyte leakage (**B**), proline content (**C**) in Chinese cabbage under heat stress. Data are shown as mean ± SD. Different lowercase letters indicate significant difference at *p* < 0.05. Ctrl, optimum conditions; HT + Ctrl, HT + GB, and HT + BABA seedlings were foliar sprayed with water, GB, and BABA, respectively, before exposure to high temperature.

Heat stress is known to intensify the generation of reactive oxygen species (ROS), which in turn cause significant increases in malondialdehyde (MDA), electrolyte leakage (EL), and proline levels [12]. In order to assess the extent of membrane damage caused by heat stress, we measured the levels of MDA and EL. As shown in Figure 2, MDA and EL remained at low levels at the normal temperature under optimum conditions. In contrast, heat caused a rapid increase in MDA and EL. Compared to the heat-stressed control, GB and BABA treatments significantly decreased MDA content by 36.61% and 49.70%, respectively (Figure 2A), and exogenous GB and BABA reduced electrolyte leakage by 24.40% and 41.81%, respectively (Figure 2B). However, the levels of MDA and EL in GB- and BABA-primed heat-stressed plants were significantly higher than those of controls under optimum conditions. Hence, the results indicate that GB and BABA priming greatly reduced the extent of membrane injury caused by heat stress, though they did not fully restore morpho-physiology to the level of the optimally grown control plants. 

Proline is known to function as an osmoprotectant that allows plants to tolerate stress [57]. Proline accumulated in the lowest concentration (15.38 µg/g FW) under optimal control conditions (Figure 2C). In contrast, heat stress alone triggered a greater than 88.10% increase in proline level relative to the control under optimum conditions. After GB and BABA treatment, proline content further increased 24.30% and 38.99%, respectively, relative to the heat-stressed control. Thus, overall, BABA seemed to be more effective in mitigating heat stress than GB, through a higher capacity of antioxidation and osmoprotection.

### 2.3. Effects of GB and BABA on the Photochemical Efficiency of Chinese Cabbage under Heat Stress

The chlorophyll fluorescence (ChlF) parameters (Table 2) provide a useful measure of photosynthetic performance in response to heat stress. For example, Fv/Fm is a sensitive indicator reflecting the maximum light energy conversion efficiency of PSII, but its decreased values may indicate stress and/or photoinhibition or indicate the downregulation of photosynthesis [11]. Furthermore, ΦPSⅡ reflects the actual primary light energy capture efficiency of the reaction center of PSII when it is partially closed, and qN reflects the part of light energy absorbed by PSII pigments and dissipated into heat instead of being used for photosynthetic electron transfer. At optimum conditions, the Fv/Fm value was 0.82, while heat stress significantly inhibited the value by 6.10%, which sharply dropped to 0.77. However, the Fv/Fm values of the GB- and BABA-primed heat-stressed plants were elevated by 1.03- and 1.04-fold, respectively, relative to the heat-stressed control. GB significantly increased the ETR, ΦPSII, and qP values by 14.92%, 21.62%, and 19.64%, respectively. BABA significantly increased the ETR, ΦPSII, and qP values by 22.18%, 27.03%, and 21.43%, respectively. Regardless of GB and BABA treatment, heat-stressed plants exhibited higher qN values than control plants under optimum conditions, implying that high temperature stress accelerated the thermal energy dissipation process in PSII. However, both GB- and BABA-treated plants significantly reduced qN by 11.11% relative to the heat-stressed control, suggesting that exogenous GB and BABA suppressed the dissipation process of the excitation of photon energy into heat in PSII. As a result, GB and BABA seem to enhance the efficiency of photochemical reaction while reducing de-excitation via heat dissipation.

### 2.4. Effects of GB and BABA on the Gas Exchange Parameters of Chinese Cabbage under Heat Stress

**Figure 3:**
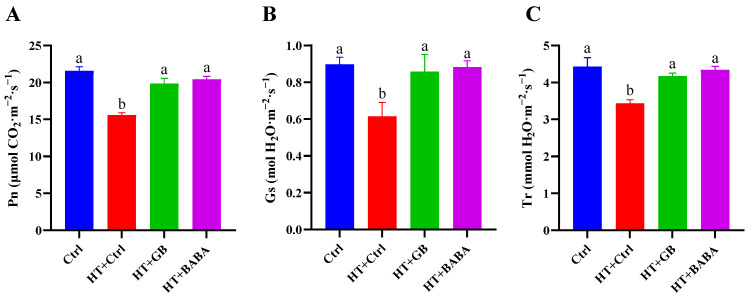
Effects of GB and BABA on gas exchange parameters in Chinese cabbage under heat stress: Pn (**A**), Gs (**B**), Tr (**C**). Data are shown as mean ± SD of five replicates. Different lowercase letters indicate significant difference at *p* < 0.05. Ctrl, optimum conditions; HT + Ctrl, HT + GB, and HT + BABA seedlings were foliar sprayed with water, GB, and BABA, respectively, before exposure to high temperature. Pn, net photosynthesis rate; Gs, stomatal conductance; Tr, transpiration rate.

In addition to ChlF, gas exchange parameters (Figure 3) are another useful means to determine photosynthetic performance in vivo. Photosynthetic rate (Pn) is a measure of the decrease in CO_2_ concentration as a function of time. Stomatal conductance (Gs) is a measure of the degree of stomatal opening that determines the rate of diffusion of CO_2_ into the leaf, or water vapor molecules out of the stomata, as a function of time. Transpiration rate (Tr) is the rate at which water moves through the plant as a function of time. The gas exchange parameters, including Pn, Gs, and Tr, were significantly decreased in heat-stressed seedlings compared to unstressed plants (Figure 3). Under heat stress, GB treatment improved levels of Pn, Gs, and Tr by 21.47%, 28.37%, and 17.64%, respectively. After BABA treatment, Pn, Gs, and Tr significantly increased by 23.65%, 30.32%, and 20.86%, respectively. As far as these gas exchange parameters are concerned, no statistically significant difference was observed between unstressed plants and seedlings treated with GB and BABA. The results are in line with a more robust photosynthetic process, as indicated by improved ChlF parameters in GB- and BABA-primed plants.

### 2.5. Effects of GB and BABA on Antioxidant Enzyme Activity in Chinese Cabbage under Heat Stress

As shown in Table 3, GB and BABA treatments increased the antioxidant enzyme activities of SOD, CAT, APX, and POD compared to the heat-stressed control. GB increased the activity of SOD, CAT, APX, and POD by 1.03-, 1.10-, 1.13-, and 1.16-fold, respectively. BABA increased the activity of SOD, CAT, APX, and POD by 1.07-, 1.14-, 1.16-, and 1.22-fold, respectively. The results are in accordance with the enhanced capacity to reduce electrolyte leakage and MDA content in GB- and BABA-primed plants.

## 3. Discussion

### 3.1. GB and BABA Promote Plant Growth by Enhancing Thermotolerance in Chinese Cabbage Seedlings

It has been documented that GB and BABA counteract the adverse effects of abiotic stress and promote the plant oxidative defense system [58,59]. In this study, chemical priming was implemented by pretreating Chinese cabbage seedlings at the third leaf stage with GB and BABA prior to applying heat stress. We found that GB and BABA pretreatment markedly increased the biomass of Chinese cabbage seedlings under heat stress. This result agrees well with previous reports that foliar application of GB and BABA boosted both fresh and dry weights of roots and shoots of maize [60,61]. GB- and BABA-primed heat-stressed plants also exhibited a significantly increased content of chlorophyll when compared to the non-primed heat-stressed plants. This is expected in light of the fact that chlorophyll content directly influences the growth and biomass of plants [5,42,60,62]. Along this line, recent studies indicated that protein hydrolysate-based biostimulants may directly stimulate carbon and nitrogen metabolism, as well as the regulation of nitrogen uptake [26,27,63]. GB was also shown to regulate phosphate homeostasis by mediating phosphate uptake and translocation [64]. Both nitrogen and phosphorus are the most important nutrients supporting the maximum yields of crops [65]. Our results, together with previously published data, suggest that exogenous GB and BABA modulates the plant physiological status so that it can promote more rapid and efficient acclimation to heat stress.

### 3.2. GB and BABA Maintain Higher Photosynthesis in Chinese Cabbage Seedlings under Heat Stress

Additional to temperature, carbon dioxide level is one of the limiting factors of photosynthesis. Reduction in photosynthesis rate was found to be associated with decreased CO_2_ absorption caused by stomatal restriction in plants exposed to heat, water, and salinity stresses [66,67]. It has been reported that high temperature stress resulted in a substantial decrease in Pn, Tr, and Gs, leading to severe inhibition of photosynthesis efficiency [5,68]. Our work shows that GB and BABA pretreatments significantly enhance these gas exchange parameters in Chinese cabbage leaves under high temperature stress. This result is consistent with previous reports that GB application via foliar spray increased Pn, Tr, and Gs levels in cotton and wheat under salt stress, and maize under drought stress [68,69,70]. Previous studies have shown that Pn can be positively correlated with thermotolerance [71,72]. Gs is known to be one of the main parameters affecting Pn and the transpiration process of plants [73,74]. It also plays an important role in plant adaptation to environmental stresses [75]. The ability to sustain leaf gas exchange under heat stress is directly correlated with thermotolerance in all plant species [76]. Thus, exogenous application of GB and BABA may improve stomatal opening in order to improve gas exchange and photosynthesis processes under high temperature stress.

Alongside gas exchange attributes, chlorophyll fluorescence measurement is a sensitive method for evaluating the degree of stress since the PSII reaction center is a key site of damage induced by abiotic stresses [77,78]. In this work, GB- and BABA-primed plants exhibited higher values of Fv/Fm, ETR, and ΦPSII than the non-primed plants (Table 2), suggesting that GB and BABA protected the activity of the photosystem from heat damage. Along this line, GB was found to be readily taken up and translocated to various organelles, including the chloroplast, thereby protecting the light-harvesting PSII from chilling stress [49]. GB and BABA priming led to increased qP and decreased qN coefficients compared to the heat-stressed control, indicating that GB and BABA promoted photochemical energy transfer and inhibited non-photochemical energy dissipation processes of PSII to sustain a high capacity of photochemical reaction despite heat stress condition. Heat stress alone increased qN, unlike other ChlF parameters, as reported previously [79]. This increase in the heat dissipation of excitation energy would result in a decrease in the efficiency of the delivery of excitation energy for PSII photochemical reactions, reflecting the lowest ChlF parameters, other than qN (Table 2). The higher qN is expected, since qN is conducive to decreases in detrimental ROS production as a photoprotective mechanism in heat-stressed plants [80]. Despite the significant difference in MDA content between GB and BABA treatments (Figure 2A), there were no significant differences in all the measured ChlF parameters between GB- and BABA-primed heat-stressed plants. Along this line, it was reported that the pattern of photochemical and non-photochemical quenching values changed via heat treatment depended on the severity of heat stress; mild heat (42 °C) stress did not significantly affect the Fv/Fm, whereas severe heat (47 °C) stress effected a 15–19% decrease [81]. Heat stress under the influence of chemical priming conditions may not necessarily affect individual photosynthetic parameters in a manner of co-linearity between the severity of heat stress and ROS level.

### 3.3. GB and BABA Increase the Activity Levels of Antioxidant Enzymes and Proline Content in Chinese Cabbage Seedlings under Heat Stress

Environmental stresses inevitably cause the accumulation of excessive reactive oxygen species (ROS). Antioxidants play a crucial role in increasing the stress tolerance of plants by scavenging ROS radicals, which can damage biomolecules, including DNA, proteins, lipids, carbohydrates, and chlorophyll pigments [12,13]. In the present study, we observed that in the control optimum condition, ROS was produced, as indicated, by MDA and EL, but in lower amounts compared to the heat stress condition; this is because ROS are produced as a normal product of cellular metabolism and are involved in signaling pathways in diverse cellular processes of unstressed cells [82]. Additionally, GB- and BABA-primed heat-stressed plants further increased the activities of SOD, APX, CAT, and POD enzymes capable of detoxifying ROS. The enhanced activities of these protective enzymes occurred in parallel with substantially reduced levels of ion leakage and MDA, used as a biomarker of oxidative stress. The higher activity levels of antioxidant enzymes would protect the integrity of the photosynthetic apparatus, and thus maintain a higher Pn under heat stress. However, despite the significant difference in MDA content between GB and BABA treatments, no significant differences in ROS-scavenging enzyme activity levels were observed between GB- and BABA-primed heat-stressed plants, both of which exhibited higher enzyme activities than the optimally conditioned control plants, containing lower levels of MDA (Figure 2A; Table 3). This could be due to the hyperbolic relationship between the rate of enzyme reaction and the substrate concentration of ROS, which typically occurs in enzyme-catalyzed reactions. Heat stress alone, i.e., without the chemical priming, resulted in much lower levels of antioxidant enzymes, indicating that uncontrolled ROS level triggered by 45 °C heat treatment caused oxidative damage to the integrity of these biomolecules, and thus decreased their activity level. Our study also shows that there were no significant differences in biomass, chlorophyll content, or ChlF parameters between GB- and BABA-primed plants under heat stress, despite their significant difference in MDA content. This implicates that the change in ROS levels retained in GB- and BABA-primed plants may not noticeably influence other morpho-physiological and biochemical attributes.

In addition, GB and BABA priming significantly increased the content of proline, which plays four major roles during stress by acting as an osmolyte, a metal chelator, an antioxidant ROS-scavenging molecule, and a redox signaling molecule [57]. Similar results were previously obtained for wheat, oat, and maize under drought stress [61,83,84]. GB is not only an osmolyte, it is also a zwitterion, and can thus interact with both hydrophilic and hydrophobic domains of protein complexes and membranes [29]. These attributes enable GB to stabilize and maintain the structural and functional integrity of cellular molecules [28,30,85]. BABA is widely known for inducing plant resistance to a broad spectrum of biotic and abiotic stresses by upregulating antioxidant enzymes, pathogenesis-related proteins, and molecular chaperones [44]. Hence, GB and BABA appear to play a prominent role in reducing oxidative damage triggered by heat stress through antioxidation and osmoprotection, thereby promoting the healthy growth of *B. rapa* seedlings.

Our results suggest that GB and BABA enhance plant functionality at the whole plant level by maintaining tolerable levels of oxidative stress in *B. rapa* seedlings under heat stress. However, their priming effects might differ depending on the stages of growth and development. It has been reported that phytohormones play a major coordinated role in regulating photosynthesis and protecting PSII damage under various stress conditions [3,19,74]. Adaptation to heat stress entailed the induction of heat shock proteins, active oxygen species, and salicylic acid and abscisic acid signaling pathways in *Arabidopsis* [86]. The susceptibility to heat stress in plants varies with the stage of plant development [76]. In this respect, further studies on the impacts of exogenous GB and BABA on phytohormone levels in *B. rapa* plants at different stages of growth and development are necessary for a more comprehensive understanding of thermotolerance processes at the molecular, physiological, and metabolic levels, thereby providing a new perspective for the increased sustainability of Chinese cabbage cultivation in high temperature climates.

## 4. Materials and Methods

### 4.1. Plant Materials and Treatments

Chinese cabbage (*Brassica rapa* L. ssp. *pekinensis*) variety of ‘Beijing No.3’ was used for the experimental plant material in the 2021 growing season in Zhejiang A&F University. Seeds were surface-sterilized in sodium hypochlorite solution, and then allowed to germinate in pots (7 × 7 × 7 cm) containing a soil mix of peat, vermiculite, and perlite at a 3:2:1 ratio. The plants were grown under long-day conditions of 16 h photoperiod, 65% relative humidity, and 600 μmol∙m^−2^∙s^−1^ maximum light intensity at 24 °C during the day and 8 h at 22 °C during the night. On the basis of the preliminary experiments determining optimum concentrations, 15 mM glycine betaine (GB, Shanghai Yuanye Bio-Technology Co., Ltd., Shanghai, China) and 0.2 mM β-aminobutyric acid (BABA, Shanghai Yuanye Bio-Technology Co., Ltd., Shanghai, China) were sprayed onto the foliage at the third leaf stage daily for 5 days. Thereafter, the seedlings were exposed to air temperature of 45 °C in 16 h light/35 °C in 8 h dark for 5 days. They were watered every day without fertilizer application. After 5 days of heat treatment, the leaf samples were harvested, quickly transferred to liquid nitrogen, and transferred to a freezer set at −80 °C before use.

### 4.2. Measurement of Growth Parameters

The seedlings of Chinese cabbage treated with GB or BABA were photographed with a Nikon D 5300 camera, and then the length, width, plant height, and fresh weight of the largest leaves were measured. Hypocotyl diameters were measured with a digital vernier caliper. Finally, the samples were placed in envelopes and dried at 75 °C, and the dry weights were measured using an electronic balance.

### 4.3. Measurement of Gas Exchange Parameters

Gas exchange parameters were determined using an LI-6800 portable photosynthesis system (LI-6800F; LI-COR, Beijing Ecotek Technology Co., Ltd., Beijing, China) on a sunny morning (9:00–12:00 A.M.). Irradiance level of light was set at 800 μmol photons·m^−2^·s^−1^. CO_2_ concentration was set at 380 μmol·mol^−1^ at 25 °C air temperature and 60% relative humidity. The net photosynthetic rate (Pn), stomatal conductance (Gs), and transpiration rate (Tr) of Chinese cabbage leaves were measured.

### 4.4. Measurement of Chlorophyll Fluorescence Parameters

Chlorophyll fluorescence was measured using a portable spectrometer (PAM-2500, Heinz Walz GmbH, Pfullingen, Germany) on the largest leaf. The detached leaf segments were clipped in the middle using dark leaf clips for 20 min at 25 °C. The maximum quantum efficiency of PSII reaction centers, as the ratio of variable to maximum fluorescence (Fv/Fm), was measured on the adaxial leaf surface immediately after dark adaptation, and calculated using the formula Fv/Fm = (Fm − Fo)/Fm, where Fo is the minimum fluorescence yield at open PSII reaction centers of a dark-adapted leaf sample, and Fm is the maximum fluorescence yield at closed PSII reaction centers after a suturing light pulse of a dark-adapted leaf sample [11,87]. Measurement of the minimum intrinsic fluorescence (Fo′) and maximum fluorescence yield (Fm′) of a light-adapted sample, as well as the steady-state level of fluorescence yield immediately before the saturating flash (Ft), revealed the actual quantum efficiency of PSII (ΦPSII), non-photochemical chlorophyll fluorescence quenching of PSII (qN), and photochemical chlorophyll fluorescence quenching (qP), using the following formulas: ΦPSII = (Fm′ − Ft)/Fm′; qN = 1 − (Fm′ − Fo′)/(Fm − Fo); qP = (Fm′ − Ft)/(Fm′ − Fo′). The electron transport rate of PSII (ETR) was determined according to the method of [88].

### 4.5. Chlorophyll Content

A chlorophyll meter SPAD (Soil Plant Analysis Development)-502Plus (Konica Minolta Sensing, Inc., Tokyo, Japan) was used to determine the chlorophyll (Chl) content at a position 2/3 of the distance from the leaf base of the uppermost fully expanded leaves.

### 4.6. Electrolyte Leakage, and MDA (Malondialdehyde) and Proline Contents

Electrolyte leakage was measured using a Model DDSJ-308F conductivity meter (Leici, Shanghai, China).

The content of MDA was determined using a slightly modified thiobarbituric acid (TBA) method [89]. Briefly, 0.5 g of fresh leaf was pulverized in 5% trichloroacetic acid using prechilled mortar and pestle, and the mixture was centrifuged. The supernatant, mixed with an equal volume of 0.67% TBA, was incubated in a boiling water bath for 10 min and cooled at room temperature. Absorbance of the sample was then measured at 450, 532, and 600 nm using a spectrophotometer. The MDA content was calculated using the equation (μM) = 6.45 × (A_532_ − A_600_) − 0.56 × A_450_, and expressed in nmol·g^−1^ FW.

Acidic ninhydrin colorimetric assay [3] was used to determine proline content from 0.5 g of fresh leaf samples pulverized in 3% sulphosalicylic acid. Absorbance of the sample was measured at 546 nm with an ultraviolet spectrophotometer (Shimadzu UV-1800, Tokyo, Japan).

### 4.7. Antioxidant Enzyme Activity Assays

Activities of superoxide dismutase (SOD), peroxidase (POD), catalase (CAT), and ascorbate peroxidase (APX) were determined using the kits R21883, R30312, R21885 (Shanghai Yuanye Bio-Technology Co., Ltd., Shanghai, China), and A123-1-1 (Nanjing Jiancheng Bioengineering Institute, Nanjing, China), respectively, according to their operating instructions. Specific enzyme activity was expressed as U·g^−^^1^ FW·min^−1^.

### 4.8. Statistical Analyses

For all treatments, five biological replications were performed. The results were expressed as mean ± standard error, and GraphPad Prism 9.0.0. software was used to draw the graphs and analyze the data. All data were subjected to analysis of variance for a factorial experiment in a completely randomized design. Statistically significant differences between means were determined at *p* < 0.05 using Tukey’s HSD (honestly significant difference) test.

## 5. Conclusions

**Figure 4:**
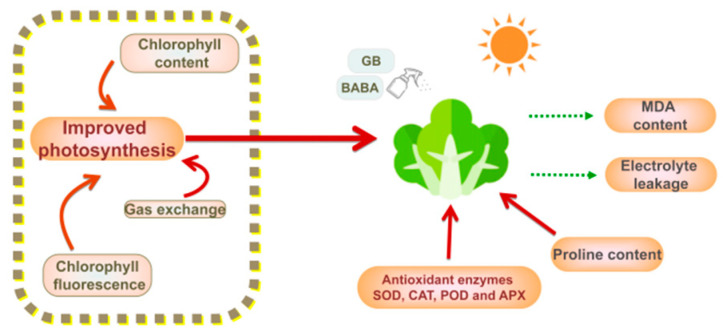
Schematic summary of GB- and BABA-primed physiological status for the preadaptation to heat stress in Chinese Cabbage seedlings. The solid arrows represent enhancement, whereas the dotted arrows represent inhibition.

In the present study, significantly improved changes in morpho-physiological and biochemical attributes were observed in GB- and BABA-primed *B. rapa* seedlings under heat stress. This entailed the coordinated increases in biomass, chlorophyll and proline contents, antioxidant enzyme activities, chlorophyll fluorescence, and gas exchange parameters in the seedlings primed with GB and BABA (Figure 4). As a result, GB and BABA, which can easily pass through the cell membrane, may offer a new integrated strategy for ecofriendly and sustainable management of Chinese cabbage to combat heat stress through higher photosynthesis performance, osmoprotection, and antioxidant enzyme activity. Functional genomics, coupled with proteomics and metabolomics, will provide more detailed insights into thermotolerance processes primed by GB and BABA at the transcriptional and post-transcriptional levels.

## Figures and Tables

**Table 1:** Effects of GB and BABA on plant growth of Chinese cabbage under heat stress.

Treatment	Fresh Weight (g)	Dry Weight (g)	Leaf Length (cm)	Leaf Width (cm)	Plant Height (cm)	Hypocotyl Diameter (mm)	SPAD
Ctrl	5.78 ± 0.24 ^a^	0.59 ± 0.02 ^a^	17.47 ± 0.28 ^a^	7.40 ± 0.14 ^a^	17.97 ± 0.29 ^a^	3.02 ± 0.02 ^a^	44.57 ± 0.38 ^a^
HT + Ctrl	3.51 ± 0.17 ^c^	0.33 ± 0.02 ^c^	11.63 ± 0.35 ^c^	4.67 ± 0.31 ^c^	12.60 ± 0.36 ^c^	2.25 ± 0.06 ^c^	29.13 ± 0.49 ^c^
HT + GB	4.42 ± 0.16 ^b^	0.45 ± 0.01 ^b^	14.59 ± 0.36 ^b^	5.06 ± 0.24 ^c^	14.79 ± 0.32 ^b^	2.41 ± 0.06 ^b^	31.97 ± 0.33 ^b^
HT + BABA	4.72 ± 0.10 ^b^	0.48 ± 0.01 ^b^	15.83 ± 0.32 ^b^	5.93 ± 0.29 ^b^	15.07 ± 0.33 ^b^	2.52 ± 0.05 ^b^	33.63 ± 0.25 ^b^

Note: Ctrl, optimum conditions; HT + Ctrl, HT + GB, and HT + BABA seedlings were foliar sprayed with water, GB, and BABA, respectively, and then exposed to high temperature. Each value represents the mean ± SD deviation. Different lowercase letters of the same column indicate significant difference at *p* < 0.05.

**Table 2:** Effects of GB and BABA on the chlorophyll fluorescence parameters under heat stress.

Treatment	Fv/Fm	ETR	ΦPSII	qN	qP
Ctrl	0.82 ± 0.04 ^a^	40.18 ± 1.03 ^a^	0.55 ± 0.79 ^a^	0.39 ± 0.01 ^ab^	0.76 ± 0.05 ^a^
HT + Ctrl	0.77 ± 0.04 ^c^	31.70 ± 0.13 ^c^	0.37 ± 0.01 ^c^	0.54 ± 0.03 ^b^	0.56 ± 0.02 ^c^
HT + GB	0.79 ± 0.02 ^b^	36.43 ± 0.91 ^b^	0.45 ± 0.03 ^b^	0.48 ± 0.05 ^a^	0.67 ± 0.03 ^b^
HT + BABA	0.80 ± 0.04 ^b^	38.73 ± 0.86 ^b^	0.47 ± 0.06 ^b^	0.48 ± 0.04 ^a^	0.68 ± 0.01 ^b^

Note: Each value represents the mean ± SD deviation of five replicates. Different lowercase letters of the same column indicate significant difference at *p* < 0.05. Ctrl, optimum conditions; HT + Ctrl, HT + GB, and HT + BABA seedlings were foliar sprayed with water, GB, and BABA, respectively, before exposure to high temperature. Fv/Fm, ratio of variable to maximum fluorescence after dark adaptation that represents the maximum photosynthetic quantum yield of PSII; ETR, electron transport rate (μmol·m^−2^‧s^−1^) of PSII; ΦPSⅡ, actual quantum yield of PSII; qN, non-photochemical quenching coefficient of ChlF; qP, photochemical quenching coefficient of ChlF.

**Table 3:** Effects of GB and BABA on antioxidant enzyme activity in Chinese cabbage under heat stress.

Treatments	Antioxidant Enzyme Activity (U·g^−1^ FW)			
	SOD	APX	CAT	POD
Ctrl	205.26 ± 0.52 ^c^	0.60 ± 0.31 ^b^	248.55 ± 0.21 ^c^	27.23 ± 1.39 ^c^
HT + Ctrl	255.31 ± 0.32 ^b^	0.56 ± 0.13 ^c^	333.44 ± 0.12 ^b^	32.29 ± 1.45 ^b^
HT + GB	261.54 ± 0.42 ^a^	0.63 ± 0.25 ^a^	367.89 ± 0.14 ^a^	37.58 ± 1.61 ^a^
HT + BABA	272.33 ± 0.49 ^a^	0.65 ± 0.26 ^a^	381.61 ± 0.11 ^a^	39.36 ± 1.63 ^a^

Note: Data are shown as mean ± SD of five replicates. Different lowercase letters indicate significant difference at *p* < 0.05. Ctrl, optimum conditions; HT + Ctrl, HT + GB, and HT + BABA seedlings were foliar sprayed with water, GB, and BABA, respectively, before exposure to high temperature. SOD, superoxide dismutase; APX, ascorbate peroxidase; CAT, catalase; POD, peroxidase.

## Data Availability

The data presented in this study are available in this manuscript.
